# Potential contribution of aspirin to cancer control programmes

**DOI:** 10.3332/ecancer.2008.100

**Published:** 2008-11-12

**Authors:** G Morgan

**Affiliations:** Welsh Aspirin Group Secretary, 41 Fforrd Beck, Gowerton, Swansea, Wales SA4 3GE, UK

## Abstract

Chemoprevention describes the potential of chemicals to intervene and block multi-stage carcinogenesis. Aspirin (acetylsalicylate) is showing cancer chemopreventive potential and the medicine has public health potential given that low doses also reduce the risk of cardiovascular events by up to 30%. Whilst recognizing that aspirin has undesirable effects, such as increasing the risk of stomach bleeding, perhaps the medicine may compliment other cancer control programmes such as screening and lifestyle measures. Furthermore, perhaps the cancer chemopreventive potential of aspirin might be mediated, at least in part, by salicylate, which is present in fruits and vegetables. Salicylate might, therefore, be considered to be a nutraceutical. Furthermore, there are a number of matters that arise including the potential for the public health field to further advocate the self-care preventive agenda, which might include aspirin. Perhaps, it is now timely for a conference on the public health potential of aspirin to be convened.

## Introduction

Chemoprevention describes the potential of pharmaceutically manufactured or naturally produced chemicals to intervene and block the complex processes of cancer formation, so called multi-stage carcinogenesis [1]. A number of chemicals with cancer chemopreventive potential have been reported in the literature, such as curcumin [2] and ginseng [3]. Some medicines in clinical use also have cancer chemopreventive properties, for example retinoids decrease the numbers of squamous cell skin carcinomas in psoriasis patients [4], and tamoxifen is used for breast cancer risk reduction in patients in remission from the disease [5].

Aspirin (acetylsalicylic acid) is an easily obtainable and inexpensive pharmaceutical medicine that is widely used to treat a number of conditions [6]. The chemical of acetylsalicylic acid was first synthesized in 1899 by Bayer Pharmaceuticals in Germany [7] and mass produced under the commercial name of aspirin. In the intervening century, aspirin has become one of the most widely used medicines around the world and it is also showing cancer chemopreventive potential [8]. The interest in this potential extends back over more than a decade and aspirin and other non-steroidal anti-inflammatory drugs (NSAIDs) were the subject of the first International Agency for Research on Cancer (IARC) scientific evaluation on cancer chemoprevention [9].

Since the IARC evaluation, selective NSAIDs have been introduced into clinical use in an attempt increase the effectiveness of this class of medicines. Some of these selective agents, such as celecoxib, offer a chemopreventive rather than surgical option against pre-cancerous adenomatous polyps in the bowel [10,11]. However, selective NSAIDs are associated with an increased risk of cardiovascular events [10,11], which raises questions about the benefit-versus-risk balance of these medicines. Such benefit-versus-risk questions would appear to make selective NSAIDs unsuitable for cancer chemoprevention in the general population.

It might be reasonably argued that of all existing and emerging cancer chemopreventive agents, aspirin has the greatest public health potential. Low-dose aspirin prophylaxis, 70–150 mg/d, reduces the risk of cardiovascular events by up to 30% [12]. Furthermore, aspirin is already well known and widely used in the general population as well as providing a benchmark to measure the effectiveness of other medicines, such as statins [13]. However, aspirin also has undesirable effects [14], most notably irritation and bleeding of the stomach. Occasionally, these undesirable effects can be serious and may even be fatal in some cases [14]. Aspirin prophylaxis against cardiovascular events therefore balances benefit and risk. The emerging evidence on aspirin and cancer chemoprevention, particularly bowel cancer, is also a relevant consideration in this benefit and risk assessment.

## Aspirin, salicylates and cancer

Randomized controlled trials (RCTs) provide evidence that aspirin reduces recurrence of either adenomatous polyps or pathological changes associated with an increased risk of bowel cancer [15,16]. The limitation to the RCTs, however, is that they only use a proxy outcome measure rather than cancer as the end point. With reference to RCTs on aspirin and cancer, there is a variation in results between trials. Two trials in the United States provided no evidence that aspirin on alternate days reduces cancer risk [17,18]. By contrast, a re-evaluation of two trials in the United Kingdom with daily aspirin found that ten years of use reduced the risk of bowel cancer by 40% [19]. Although different doses were used in the four trials, one of the US trials and one of the UK trials used similar doses of 325 and 300 mg, respectively. Furthermore, one of the US trials also had a follow-up of ten years, which raises questions about the dose and duration effect of aspirin.

Possibly, the difference in the results between the US and UK trials might be due, at least in part, to the different dosing regimes of alternate days versus daily, respectively. Possibly, daily aspirin exposure over ten years might be required to reduce the risk of bowel cancer. Furthermore, the current evidence suggests that perhaps doses of aspirin higher than those used routinely for cardiovascular event prophylaxis might be required for chemoprevention. The risk of undesirable effects from aspirin increases with both age and dose. This is an important point given that many cancers, such as bowel cancer, tend to have rising incidence in the general population with increasing age.

Perhaps, there might also be another dimension with the possible cancer chemopreventive effects of aspirin. The potential cancer chemopreventive effects might be related, at least in part, to salicylate. Humans are exposed to salicylate through eating fruit and vegetables, and it has been suggested that perhaps salicylate might be beneficial to health [20]. Salicylate has anti-oxidant effects, anti-inflammatory properties and induces programmed cell death or apoptosis in cancer cells [21]. More evidence is needed on the effect of salicylate on human health generally and cancer chemoprevention specifically. This also raises a wider issue of whether components of food can be helpful in reducing disease risk.

Nutraceuticals are a food, or part of a food, that provides medical or health benefits, including the prevention or possibly treatment of a disease [22]. The term nutraceutical is a hybrid of nutrition and pharmaceutical [23]. It has been suggested that a **‘nutraceutical a day may keep the doctor away’** and that **‘consumers are turning increasingly to food supplements to improve well being when pharmaceuticals fail’** [23]. This however raises further issues given that there is evidence that supplements of some vitamins may increase the risk of premature mortality [24]. A clear scientific definition of the criteria for vitamin status and also the introduction of a regulatory system for their supply and demand might be some of the developments taken forward.

In future, there might be more attention given to the potential value of nutraceuticals in the promotion of human health. There are numerous examples of nutraceuticals published in the literature and, for example, these have relevance to the chemoprevention of stomach [25] and prostate [26] cancers. Poly-unsaturated fatty acids (PUFAs) have anti-inflammatory effects [27] and since chronic low-level inflammation can increase the risk of some cancers [28], perhaps both PUFAs and salicylates may have cancer chemopreventive properties. As a related point, inflammation in vascular tissue also appears to be an aetiological factor in cardiovascular events [29,30].

Research therefore appears to be warranted on whether salicylate derived from fruit and vegetables has nutraceutical properties. Such research may contribute to a wider debate on dietary changes in the population. So-called ***epidemiological diet transition*** suggests that populations are eating more processed foods and less fruit and vegetables [31]. Possibly, reduced dietary intake of salicylate in the population might predispose some individuals to increased cancer risk and perhaps aspirin might counteract this deficiency. This may also provide further reasons to promote the intake of salicylate rich fruit and vegetables, such as berries and organic produce, in the general population. However, it also needs to be recognized that chemoprevention might be only one element of cancer control programmes, alongside other measures such as screening and lifestyle approaches.

## Other cancer control programmes

Screening is one of the important cancer control programmes. In screening, individuals are asked a question or offered a test to identify those who might be helped by further investigation. Screening programmes include breast and bowel cancers. Taking the former, a review of breast cancer screening by the evidence-based body, the Cochrane Collaboration, suggested that the programme of mammography in women over the age of 50 years lowers risk of breast cancer mortality by about 15% [32]. However, the review also highlighted that there are some undesirable aspects of breast cancer screening for some women. This includes inaccurate results leading to unnecessary treatment for some women. The review called for women who are invited to breast cancer screening to be **‘fully informed of both benefits and harms’**. This statement also appears to be pertinent to aspirin prophylaxis since the medicine also has benefits and risks. There is also some promising evidence that aspirin may also reduce the risk of breast cancer [33] although, similar to bowel cancer, low doses on alternate days may not have chemopreventive effects [34].

One of the limitations to the breast cancer screening programme is that younger women at increased risk of the disease are not routinely included [35]. Family history of breast cancer is a strong predictor of disease development and is one of the factors used in compiling at risk registers for women. Such registers can then be used to follow up women at increased risk of breast cancer and in addition, primary care has been highlighted as a potential setting to identify those who may have hereditary predisposition to the disease, such as BRCA mutation carriers [36].

With respect to the bowel cancer screening programme, this includes the faecal occult blood test (FOBT). Similar to the breast cancer programme, a Cochrane Collaboration review has highlighted the potential undesirable aspects of inaccurate results and unnecessary treatment [37]. Bowel cancer screening programmes vary in their delivery, but they also usually include individuals over the age of 50 years. In addition, younger high-risk patients who are genetically predisposed to bowel cancer are usually identifiable, and in the United Kingdom they are often registered with a Regional Genetics Centre. High-risk patients include those with genetic conditions, including hereditary non-polyposis colorectal cancer (HNPCC) and familial adenomatous polyposis (FAP). HNPCC patients usually receive surveillance of the bowel from the age of 25 years, whilst in the case of FAP, prophylactic surgery before the age of 25 years is usually recommended [38].

One of the issues with the bowel cancer screening programme relates to the screening algorithm based upon FOBT and colonoscopy for those testing positive, with perhaps radiology also contributing in future [39]. Although FOBT is a simple and non-invasive test, it has poor sensitivity and may only detect a bleeding lesion rather than cancerous changes [40]. Furthermore, colonoscopy rather than FOBT may be the most appropriate initial investigation for individuals with higher than average risk of bowel cancer [41]. In developing bowel cancer screening programmes, considerations include patient preferences, likelihood of adherence to follow up and resources available [42].

Another issue with the bowel cancer screening programme relates to uptake. For example in the UK Bowel Cancer Screening Pilot Programme, nearly 130,000 men and women aged 50–69 years were invited to participate and uptake ranged from 61% in the wealthiest areas to 37% in the poorest areas [43]. Does this mean that screening should be more intensively targeted in the areas of lowest uptake? This possibility does deserve to be considered although the UK Bowel Cancer Screening Programme is already financially constrained [44] so intensive targeting may not be a possibility within available resources.

Self-care is important [45] and perhaps consideration could be given to the potential contribution of aspirin to bowel cancer screening programmes. Individuals who have pre-cancerous lesions removed from the bowel in the programme might at least be offered the evidence on the benefits and risks of low-dose aspirin given that this might reduce the risk of subsequent pathological changes associated with bowel cancer development. There is an important related dimension to this. Many individuals at the age of 50 have a risk of cardiovascular events in which considering taking low-dose aspirin prophylaxis may be a reasonable option [46,47], although this possibility has been debated [48]. It may be recognized, however, that cardiovascular events and cancer are the biggest causes of disease, disability and death in the population, and so increased use of aspirin might confer considerable public health benefits if targeted appropriately [49].

It may be reasonably concluded that cancer screening programmes do not represent a complete solution to cancer control and do have negative aspects as well. So in addition to screening programmes, there are also lifestyle approaches to cancer control. For example the deleterious effects of smoking on health and the importance of cessation have been well documented, especially within the context of deprivation and low socio-economic status in which the highest smoking rates are found [50]. Gender may also play an important role as well, since there is suggestive evidence that women might be more susceptible to developing smoking-related illnesses compared with men [51]. Whilst smoking cessation programmes are important, other measures such as advertising bans on tobacco products might also contribute to cancer control programmes [52].

Alcohol consumption is sometimes closely associated with smoking [53] and even moderate alcohol consumption may increase the risk of some cancers, such as breast. This increased risk, however, conflicts with health promotion advice on moderate alcohol consumption for the cardiovascular benefits [54]. Cancer control programmes therefore need to find a balance between the benefits and risks of alcohol consumption, which raises wider social considerations.

Diet is also important, and IARC are co-ordinating the European Prospective Investigation into Cancer and Nutrition study across ten countries. This includes more than half a million participants of whom 70% are men between the ages of 35 and 70 years. This study shows the impact of diet on cancer risk [55], for example, vegetables, which contain salicylate, appear to reduce the risk of stomach cancer. In addition, red meat appears to increase the risk of bowel cancer whilst fish, which contain PUFAs, may decrease the risk of the disease. Furthermore, there is evidence that obesity is a risk factor for cancer as well, for example breast and bowel cancers in post-menopausal women [56]. This highlights the potential importance of both diet and exercise in reducing cancer risk through a process of weight control.

However, two publications, of relevance to weight control, illustrate the complexity of lifestyle approaches to reducing the risk of cancer. In November 2007, a World Cancer Research Fund (WRCF) report set out ten rules for preventing cancer [57]. One of these rules was having a Body Mass Index (BMI) of 25 or lower. Shortly after the publication of WRCF report, the US Centers for Disease Control and Prevention (CDC&P) also presented cancer control recommendations [58]. This report provided evidence that being underweight and obesity carry increased risks of mortality, albeit from different causes. Furthermore, it was suggested that being overweight, defined as having a BMI of between 25–30, was associated with decreased mortality overall and was not associated with mortality from either cancer or cardiovascular causes.

With both of the WRCF and CDC&P reports, the media coverage was extensive and to some extent contradictory. This contradiction appears to be consistent with the comment of the Public Library of Science Medical Editors, who stated: **‘It is not always easy for the public to determine what is best given the barrage of information forced on them every week’** [59]. Lessons from other medical issues also highlight the need for health professionals to provide the general public with clear and consistent information. For example, in 1998, a controversial study linked measles, mumps and rubella (MMR) with autism [60], and this was widely reported in the media. Since 1998, the uptake of MMR vaccination has declined and a study on the reasons has suggested that inadequate information from healthcare professionals was an important contributory factor [61].

If lifestyle approaches to cancer control are to be fully maximized then it is important for the general public to be given clear and consistent information by healthcare professionals. This is pertinent to aspirin. If the general public is to be given clear and consistent information on ways to reduce the risk of cancer, at what stage is the emerging evidence on aspirin systematically put into the public domain to allow individuals to make their own informed decisions on whether or not to take aspirin? This is a far reaching question that deserves to be debated further, particularly since the information on aspirin and cancer is already being reported in the media with differing levels of accuracy.

For the purposes of comprehension, the use of vaccines to reduce cancer risk also merits brief mention to illustrate the broader context of cancer control programmes. For example, the human papillomavirus (HPV) is a sexually transmitted infection, which causes genital warts and some cancers, most notably cervical cancer. HPV vaccination programmes to reduce the risk of cervical cancer may be seen as a complement rather than an alternative to the screening programme for this disease [62]. Although some issues still need to be given further consideration with HPV vaccination, including the duration of protection [63], the broader point with wider application is that different cancer control approaches can be combined.

## From preventive self-care to public health policy

The population is ageing and with individuals living longer, service provision is becoming increasingly community based [64] and multi-disciplinary [65] in some countries. In addition to the changing patterns of service provision, self-care is important [66], and this has the potential to improve the management of long-term conditions whilst also taking account of patient safety issues [67]. The potential of self-care, however, also extends to prevention as well as self-examination for the early identification of testicular cancer. It has also been stated that an **‘ounce of prevention is worth a pound of cure’** and one way to achieve this may be to **‘offer financial incentives to people for healthy behaviour’** [68]. This is a controversial suggestion that could have far reaching implications for the delivery of public health policy.

Public health may be considered as improving and protecting the health of groups of people, or populations, rather than treating individual patients. Public health has a proud tradition [69], for example in 1848, the Public Health Act for England and Wales [70] and Sir Edwin Chadwick, the driving force behind this Act, has been described as leaving a **‘monumental’** public health legacy [71]. Although some have argued that public health is in decline [72,73], the framework for the delivery of public health continues to evolve [74]. So perhaps there is an opportunity for the field of public health to further advocate the self-care preventive agenda, which might include aspirin. This could be progressed through collaborative working, which has been suggested as an important leadership issue in public health [75], for example in the so-called communities of practice with other professional groups [76].

It is reasonable to acknowledge that there is a considerable amount of knowledge on lifestyle risk factors for disease. For example a cohort study of 20,000 men and women aged 45–79 years in east England is part of the IARC European Prospective Investigation on Cancer and Nutrition study. The study has suggested that regular exercise, 1–14 units of alcohol per week (unit = glass of wine or half pint of beer), eating five servings of fruits and vegetables per day and not currently smoking might prolong life by 14 years [77]. The public health potential of aspirin might be viewed as complement rather than an alternative or competitor to these beneficial lifestyle factors. However, the potential increased use of the medicine still raises a number of debates and ethical considerations [78], some of which may be presented to the public through the media.

In balance to the two illustrations of media interest in health issues presented previously, it may be acknowledged that media involvement can be beneficial. For example, in Wales, the bowel cancer screening programme was launched at the end of October 2008. Television advertising was used as one of the methods to raise awareness of both the disease and the screening programme. Perhaps the wider lesson is that close working between media and health professionals can usefully disseminate accurate information to the benefit of the targeted population.

## Conclusion

Currently, there is a trend towards an increase in life expectancy in the population, which appears to be attributable to improvements in medicine, public health and agriculture [79]. The two biggest causes of disease, disability and death in the population are cardiovascular disease and cancer, and increased aspirin use might reduce their burden. The increased use of aspirin may in future form part of cancer control programmes and the increased use of the medicine in the population may confer considerable public health benefits. These benefits could lead to a further shift towards preventive self-care, which might in turn help reduce the pressure on healthcare services. Indeed, even the iconic National Health Service in the United Kingdom has limitations in caring for the population over the age of 50 years [80]. So there might be considerable population need, defined by health economists as capacity to benefit from treatment [81], for increased aspirin use. However, the potential benefits from increased aspirin use in the population need to be balanced by the undesirable effects of the medicine. Perhaps, it is time for another conference on the public health potential of aspirin to be convened [82] so that this important matter can be given further consideration with a view to appropriate policy responses being developed in accordance with the evidence.

Part of this evidence base will need to take account of the existing levels of aspirin use within the population [83], which may require some surveys to be undertaken in some countries.

In addition, the natural exposure of nutraceutical salicylate through the diet might also contribute to cancer control programmes in future and further research is required.
